# Molecular Dynamics Simulation of the Complex of PDE5 and Evodiamine

**DOI:** 10.3390/life13020578

**Published:** 2023-02-18

**Authors:** Ayame Kobayashi, Motokuni Nakajima, Yoh Noguchi, Ryota Morikawa, Yukiko Matsuo, Masako Takasu

**Affiliations:** 1 School of Life Sciences, Tokyo University of Pharmacy and Life Sciences, Tokyo 192-0392, Japan; 2 The Institute of Statistical Mathematics, Tokyo 190-8562, Japan; 3 School of Pharmacy, Tokyo University of Pharmacy and Life Sciences, Tokyo 192-0392, Japan

**Keywords:** molecular dynamics simulation, PDE5, evodiamine, Alzheimer’s disease, natural product

## Abstract

Alzheimer’s disease is an irreversible neurological disorder for which there are no effective small molecule therapeutics. A phosphodiesterase 5 (PDE5) inhibitor is a candidate medicine for the treatment of Alzheimer’s disease. Rutaecarpine, an indole alkaloid found in *Euodiae Fructus*, has inhibitory activity for PDE5. *Euodiae Fructus* contains more evodiamine than rutaecarpine. Therefore, we performed molecular dynamics simulations of the complex of PDE5 and evodiamine. The results showed that the PDE5 and (−)-evodiamine complexes were placed inside the reaction center compared to the case of PDE5 and (+)-evodiamine complex. The binding of (−)-evodiamine to PDE5 increased the root-mean-square deviation and radius of gyration of PDE5. In the PDE5 with (−)-evodiamine complex, the value of the root-mean-square fluctuation of the M-loop, which is thought to be important for activity, increased. This result suggests that (−)-evodiamine may have inhibitory activity.

## 1. Introduction

**Figure 1:**
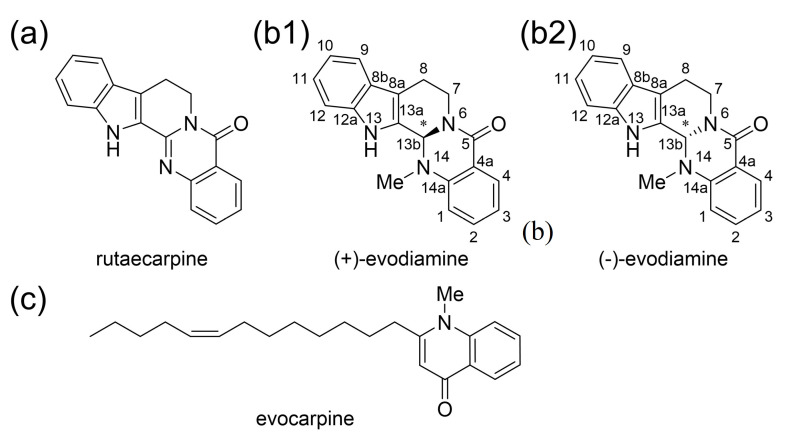
The active compounds of *Euodiae Fructus*. (**a**) Rutaecarpine and (**b**) evodiamine, indole alkaloids isolated from *Euodiae Fructus*. (**c**) Quinone alkaloid isolated from *Euodiae Fructus*.

*Euodiae Fructus* is an important crude drug and comprises Goshuyuto, a Kampo medicine clinically used for chronic headaches and migraine attacks [1]. The active compounds of *Euodiae Fructus* are known as indole alkaloids and quinone alkaloids such as rutaecarpine, evodiamine, and evocarpine (Figure 1a,b) [2]. It is known that the amount of evodiamine in *Euodiae Fructus* is about the same as the amount of rutaecarpine [2]. Evodiamine has one chiral center. *Euodiae Fructus* contains more (+)-evodiamine than (−)-evodiamine [3].

Alzheimer’s disease (AD) is characterized by neuronal loss, senile cell groups, and intracellular neurofibrillary changes that cause memory loss [4]. Donepezil [5] and Galantamine [6], acetylcholinesterase inhibitors, and Memantine [7], an N-methyl-D-aspartate (NMDA) receptor antagonist, are known as drugs for AD. In general, it is suggested that AD begins as a disorder produced at least in part by amyloid-β (Aβ). The cAMP-responsive element binding protein (CREB) is an important factor involved in memory [8,9], and phosphorylation of this protein is critical for CREB activity. A typical protein that activates CREB is protein kinase A (PKA), which is known to be activated by cAMP and subsequently phosphorylates CREB [10]. Aβ is known to cause synaptic dysfunction by inhibiting CREB phosphorylation [11].

**Figure 2:**
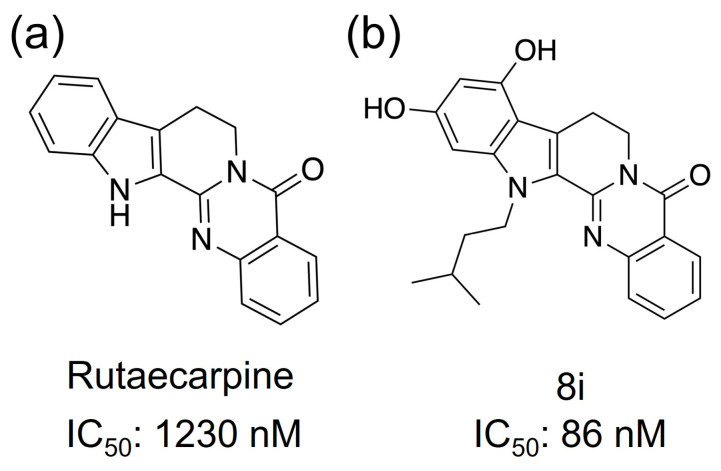
Compounds that have shown PDE5 inhibitory activity. (**a**) Rutaecarpine and (**b**) 8i, which is an analog.

Phosphodiesterase (PDE) is an enzyme that hydrolyzes cyclic phosphate diesters of cAMP and cGMP. Therefore, when the function of PDE is inhibited, blood levels of cAMP or cGMP increase. PDE inhibitors modulate signaling pathways by increasing levels of cAMP or cGMP and activate CREB via PKA [12,13]; activation of CREB has been reported to promote neurotransmission, synaptic plasticity, and memory formation [14]. mRNA of the PDE5, one of the family proteins of PDE, has been detected in the human cortex, hippocampus, and striatum, and PDE5 inhibitors have been reported to cross the blood–brain barrier and promote vasodilation by inhibiting PDE in neurons and glial cells [15,16]. The phosphodiesterase 5 (PDE5) protein inhibitor is a candidate for the cure for AD [17,18]. Xian-Feng and coworkers synthesized a series of novel PDE5 inhibitors based on rutaecarpine and conducted studies on PDE5 inhibitory activity and in vivo memory and cognitive function [19]. The IC50 (half maximal inhibitory concentration) value of rutaecarpine (Figure 2a) showed PDE5 inhibition at approximately 1230 nM, indicating that it is a promising candidate as an inhibitor. Compound 8i, a rutaecarpine derivative (Figure 2b), was found to be a strong inhibitor with an IC50 value of about 86 nM.

The C-terminal catalytic domain of PDE5 has four subdomain active sites (Q pocket, H pocket, L region, and M site) [20]. The Q pocket is a highly conserved region in the PDE5 catalytic domain and contains the common Gln817, Phe820, Val782, and Tyr612 amino acid residues. The conserved Gln817 amide group forms a bidentate hydrogen bond with the guanine group of cGMP, stabilizing the cGMP structure of the active site. Other residues in this pocket form hydrophobic interactions with cGMP. The H pocket is composed of variable hydrophobic residues that contribute to the inhibitor selectivity of PDE5. The M site contains ions that bind to metal-binding residues common to PDE5, thus stabilizing the structure and activating the hydroxide of the active site. The active site is activated to promote hydrolysis of cGMP and interacts directly with cGMP substrates and indirectly with existing inhibitors. The L region is known to play a role in inhibitor binding by changing its conformation from closed to open [21].

**Figure 3:**
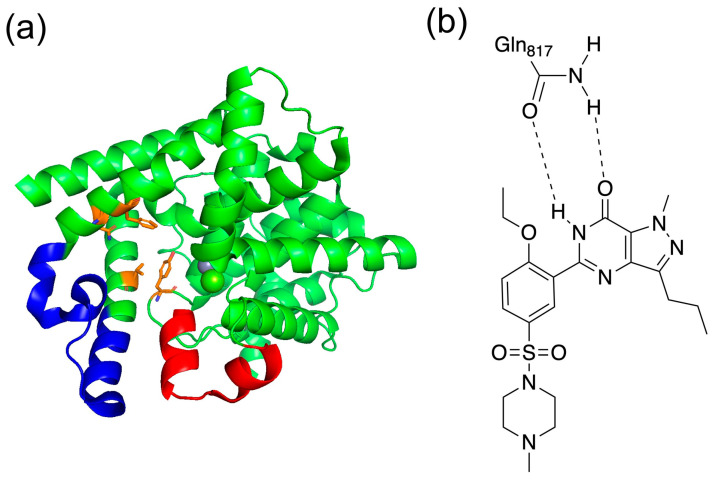
(**a**) Structure of PDE5 and (**b**) binding with sildenafil. In (**a**), the red area is the H-loop, the blue area is the M-loop, and orange is the residues of Q pocket. In (**b**), the bidentate hydrogen bond is shown between Gln817 in PDE5 and sildenafil.

Additionally, the C-terminal catalytic site of PDE5 has two characteristic loop structures. The unit containing residues 661–676 in PDE5 is called the H-loop, and the one containing residues 787–812 is called the M-loop (Figure 3a) [22]. These two loop structures are close to the active site and are important for the activity of the inhibitor. It has been shown that the selective inhibition of PDE5 by sildenafil, which is the best known PDE5 inhibitor, involves the binding between sildenafil and the M-loop or H-loop [20]. It has also been reported that the H-loop and M-loop structures are disordered when sildenafil was bound [22]. As shown in Figure 3b, sildenafil binds with a bidentate hydrogen bond between Gln817.

It has been shown that rutaecarpine, one of the active components of *Euodiae Fructus*, exhibits PDE5 inhibitory activity [19]. Evodiamine, also from *Euodiae Fructus*, is an alkaloid with a similar structure to rutaecarpine, but has some differences, such as a methyl group on the N14 side-chain [3]. 

We performed docking simulations of (+)-evodiamine and (−)-evodiamine with PDE5. Using each complex with smallest free energy, we carried out molecular dynamics (MD) simulations. Our results show that both evodiamines docked in the same pocket as the existing PDE5 inhibitor, and (−)-evodiamine showed the structural change in the M-loop.

## 2. Model and Methods

### 2.1. Simulation Preparation

We prepared the structure of PDE5 and its (+) or (−)-evodiamine complex. The PDE5 and evodiamine structures for our MD simulations were obtained from existing databases (PDB and Zinc20). The X-ray crystal structure of catalytic domain of human PDE5A (ID: 1XOZ) [23] was obtained as the structure of PDE5. As the structure of (+) or (−)-evodiamine, we used the three-dimensional structural data in mol2 file format (ID: ZINC898159 and ID: ZINC2031813, respectively) from the Zinc20 database [24]. The structures of each complex were created from the obtained PDE5 structure and the structure of evodiamine using SwissDock [25]. SwissDock resulted in 32 different clusters and a total of 256 structures, of which the lowest energy model was used.

### 2.2. Molecular Dynamics Simulation 

MD simulations of protein with or without the binding of evodiamine were performed using GROMACS (ver 5.1.2 [26], double precision) for 200 ns. A cubic system was constructed so that the distance between the protein and the system boundary is at least 1 nm. The total overall energy was then minimized to 1000 kJ mol^−1^ nm^−1^ by steepest descent minimization. We performed the simulation with NVT ensemble for 100 ps and NPT ensemble for 300 ps. After energy minimization, we used velocity-rescaling [27] for NVT and Parrinello–Rahman pressure coupling [28] for NPT. The simulation is performed under periodic boundary conditions. Amber99SB-ILDN [29] was used for the force field of PDE5 and ions, and general AMBER force field (gaff) [30] was used for the force field of evodiamine. We used TIP3P as our water model. The parameters for MD simulations are shown in Table 1. Zn^2+^ and Mg^2+^ are the metal ions of PDE5, while Na^+^ is the counter ion to adjust the charge of the system. Since PDE5 naturally resides in the cytoplasm, it does not have the same ionic properties as saline. Therefore, we chose a total charge of zero for the simulation rather than physiological conditions. The size of the box is the size after the short simulation with NPT ensemble.

### 2.3. Quantum Mechanical Calculation

The structures of (+)-evodiamine at 1, 75, and 200 ns and (−)-evodiamine at 1 and 75 ns obtained by MD simulations were structurally optimized by quantum mechanical (QM) calculations with Gaussian16 Revision B.01 [31]. We used B3LYP [32] with 6-31G* basis set. Here, a very tight option was used for the structural optimization option. For the grid, Ultrafine was used. Up to 250 steps of optimization calculations were performed. Then, for each optimization structure, vibrational frequency was calculated under the same conditions.

### 2.4. Analysis

The radius of gyration (*R*_g_), root-mean-square deviation (RMSD), root-mean-square fluctuation (RMSF), and number of hydrogen bonds were calculated and analyzed for PDE5 and the complexes. These calculations were performed using GROMACS modules. *R*_g_ is defined by

(1) $$Rg=\sqrt{\frac{\sum_{i=1}m_{i}{r_{i}}^{2}}{\sum_{i=1}m_{i}}}$$

where $m_{i}$ is the mass of atom i, and $r_{i}$ is the position of atom i. RMSD is defined by

(2) $$\mathrm{RMSD}=\sqrt{\frac{\sum_{i=1}^{N}m_{i}{\left\{\left(r_{i}\left(t\right)-r_{i}(t_{0}\right)\right\}}^{2}}{\sum_{i=1}^{N}m_{i}}}$$

where N is the number of atoms, $m_{i}$ is the mass of atom i, $t_{0}$ is the start time of the simulation, t is time, and $r_{i}$ is the position of atom i. This calculation was performed after fitting was done using the least squares method. RMSF is defined by

(3) $${\mathrm{RMSF}}_{i}=\sqrt{\frac{1}{T-T_{0}}\sum_{t_{j}=T_{0}}^{T}{\left(r_{i}\left(t_{j}\right)-{\langle r_{i}\rangle }_{t}\right)}^{2}}$$

where t is time, $T_{0}$ is the reference time for the calculation, T is time at the end, $r_{i}$ is position of residue i, and ${\langle r_{i}\rangle }_{t}$ is position averaged over time of particle 𝑖. The relationship between RMSF and B-factor $B_{i}$ is

(4) $$B_{i}=\frac{8\pi^{2}}{3}{\left({\mathrm{RMSF}}_{i}\right)}^{2}$$

where RMSF_*i*_ is the RMSF of residue i.

**Figure 4:**
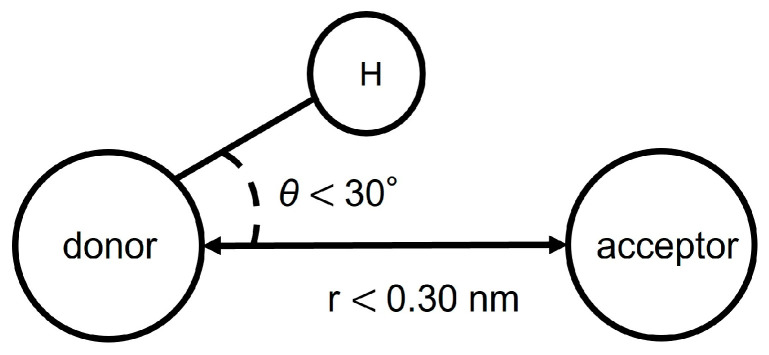
Conditions for hydrogen bonding.

The formation of hydrogen bonds was determined from the distance and angle between the donor atom, acceptor atom, and hydrogen atom. Hydrogen bonds are counted when both of the following two conditions are met (Figure 4). First, the distance between donor and acceptor is less than 0.30 nm. Secondly, the angle θ made by hydrogen, donor and acceptor is less than 30 degrees.

## 3. Results

### 3.1. Docking Simulation

**Figure 5:**
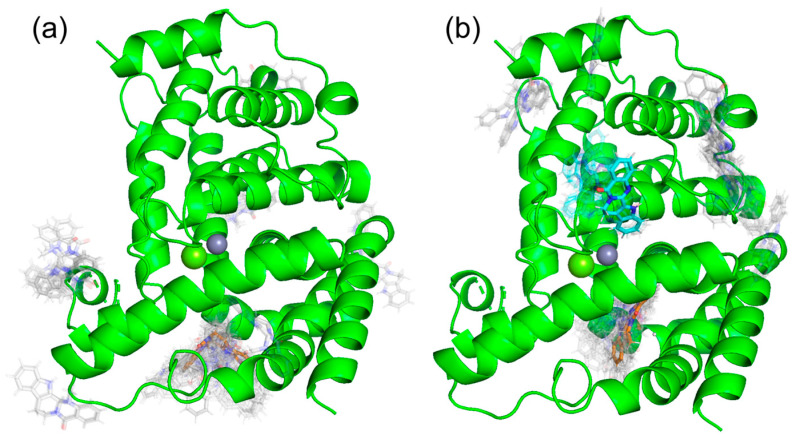
Results of docking simulation. (**a**) (+)-evodiamine, (**b**) (−)-evodiamine.

Docking simulations were performed using SwissDock shown in Figure 5. As a result, we found one docking site with a binding Gibbs free energy of ΔG = −7.568 kcal/mol, shown in orange in Figure 5a, in (+)-evodiamine. Similiarly, as shown in Figure 5b, (−)-evodiamine has a docking site with the binding free energy of ΔG = −7.568 kcal/mol, shown in orange, and a binding free energy of ΔG = −7.190 kcal/mol, shown in cyan. In both cases, the binding free energy at the orange site was the lowest, so MD simulations were performed for the orange site.

### 3.2. RMSD and R_g_

**Figure 6:**
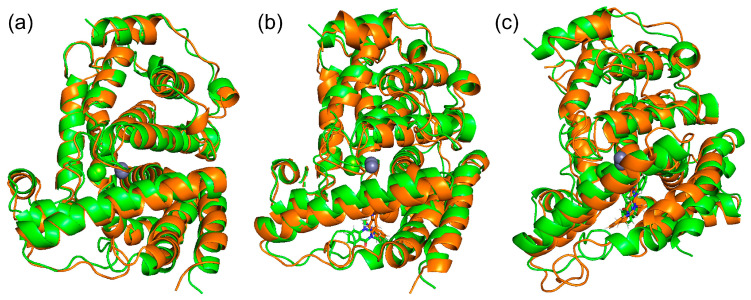
Snapshots of PDE and evodiamine complex structure for MD simulation. (**a**) Superimposed snapshots of the structure of PDE5 at 0 ns and 200 ns. (**b**) PDE5 with (+)-evodiamine complex, (**c**) PDE5 with (−)-evodiamine complex. Green: 0 ns, orange: 200 ns.

We performed MD simulations of PDE5 and PDE5 with (+)-evodiamine and PDE5 with (−)-evodiamine. The overlapping snapshots at 0 ns and 200 ns (Figure 6) showed that the M-loop of (−)-evodiamine has changed. 

**Figure 7:**
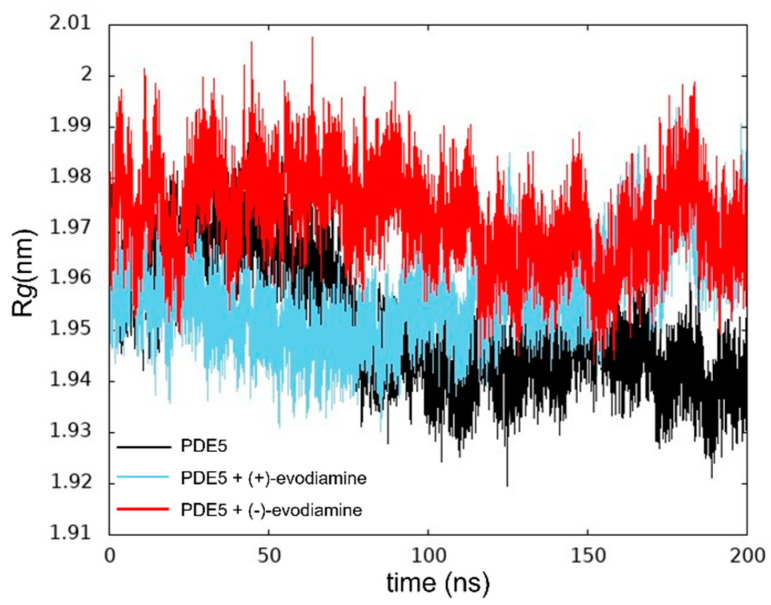
Time evolution of *R*_g_.

We calculated *R*_g_ to ensure that the protein was not unfolding. In Figure 7, we show the *R*_g_ values at 200 ns of PDE5. The changes in these values of *R*_g_ are relatively small. 

**Figure 8:**
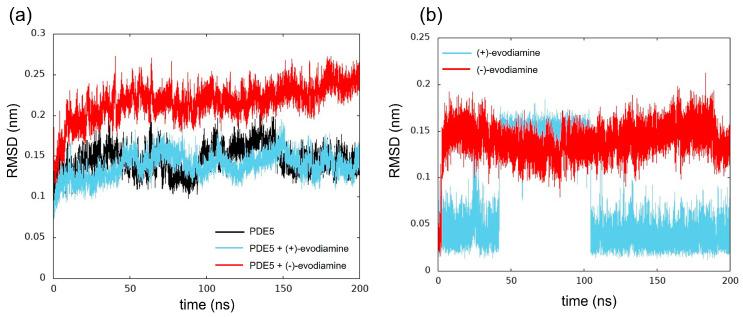
Time evolution of RMSD. (**a**) RMSD of PDE5 in each model, (**b**) evodiamine.

We computed the RMSD as shown in Figure 8. The structure of the reference was at 0 ns. In Figure 8a, we show the time evolution of RMSD for PDE5 and PDE5 complexes. The RMSD values of PDE5 with (−)-evodiamine complex were significantly different from those for PDE5 and PDE5 with (+)-evodiamine complex. The change in the RMSD values of the PDE5 with (−)-evodiamine complex became smaller after 50 ns. After 50 ns, PDE5 complexes show stable structure. 

The RMSD was calculated to investigate the conformational changes of evodiamine. In Figure 8b, we show the time evolution of RMSD for both cases of evodiamine. This result suggests that there are two states in the structure of evodiamine. The first state is a structure with an RMSD of 0.02 nm to 0.07 nm, and the second state is a structure with an RMSD of 0.10 nm or higher.

**Figure 9:**
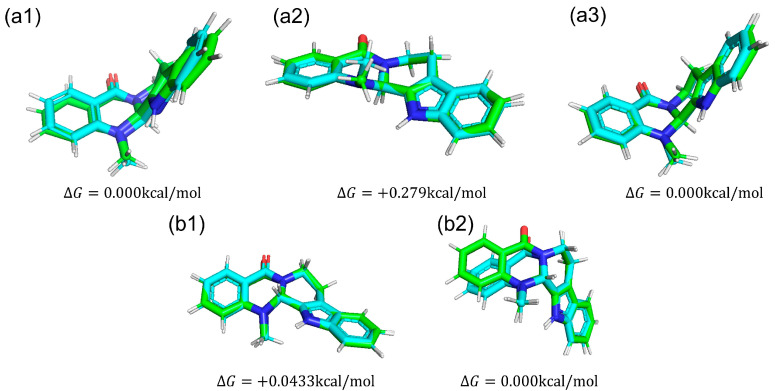
Structures of each evodiamine extracted from MD simulations (green) were optimized for QM calculations (cyan) of (+)-evodiamine: (**a1**) 1 ns, (**a2**) 75 ns, (**a3**) 200 ns. (−)-evodiamine: (**b1**) 1 ns, (**b2**) 200 ns.

We compared conformations of evodiamine. In Figure 9, we show five snapshots of evodiamine. From those snapshots, we can see that one state (Figure 9a1,a3,b2) is a tilted structure, and another state (Figure 9a2,b1) is a planar structure.

**Figure 10:**
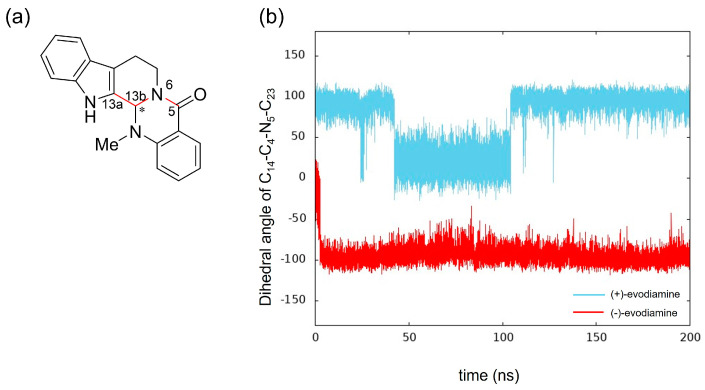
Time evolution of conformational change of (+)-evodiamine (blue) and (−)-evodiamine (red): (**a**) evodiamine C_5_-N_6_-C_13b_-C_13a_, (**b**) dihedral angle of C_5_-N_6_-C_13b_-C_13a_.

We calculated the dihedral angle consisting of C_5_-N_6_-C_13b_-C_13a_ (Figure 10a) to confirm whether this methyl group change is caused by the change in RMSD of evodiamine. The results showed that its time evolution was consistent with the change in RMSD. This indicates that the conformation of evodiamine is changing.

The results of MD simulation show a high frequency of occurrence of structures with a dihedral angle of about −100 (100) degrees (Figure 10b). To investigate the structural stability of (+)-evodiamine, the energies of their structures at 1 ns and 75 ns were calculated by quantum mechanical method. As a result, it was found that the energy of the structure at 75 ns was larger by 0.279 kcal/mol than that at 1 ns. This indicates that the structure at 75 ns is less stable than the other structure.

### 3.3. Structural Changes in the Loops of the Complex

**Figure 11:**
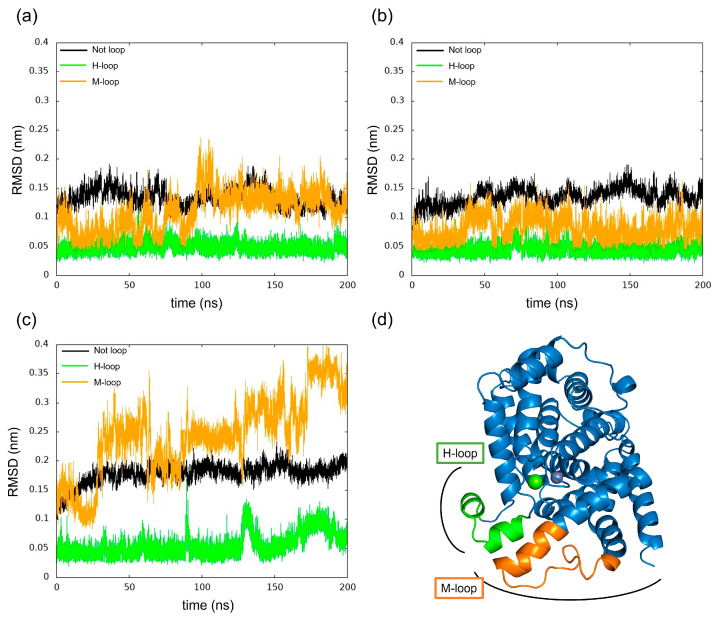
Time evolution of RMSD for H-loop and M-loop. (**a**) PDE5, (**b**) PDE5-(+)-evodiamine complex, (**c**) PDE5-(−)-evodiamine complex. (**d**) Position of H-loop and M-loop of PDE5.

We have calculated the RMSD of PDE5 and complexes separately for each loop and non-loop structure for changes in RMSD as shown in Figure 11. The results show that in each model, the change in the H-loop was small, while the M-loop in PDE5 with (−)-evodiamine complex showed large RMSD values.

### 3.4. Effect of Evodiamine Binding on Fluctuations

**Figure 12:**
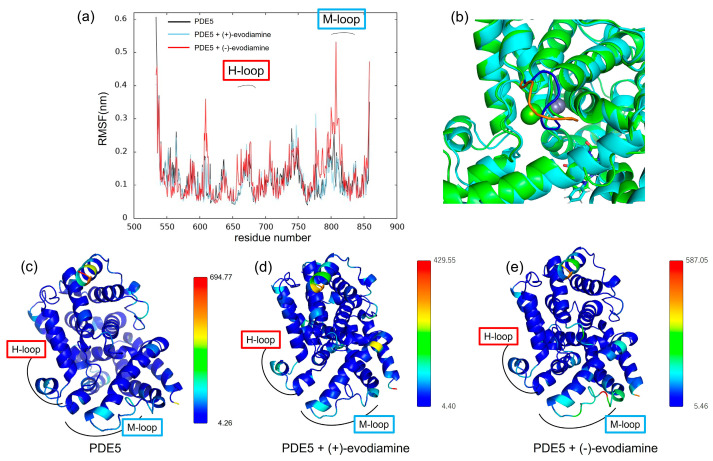
(**a**) RMSF at 50–200 ns. (**b**) Snapshot of PDE5 with (−)-evodiamine complex at 50 ns and 200 ns. The structure of PDE5 (**c**) and PDE5 with (+)-evodiamine complex (**d**) and PDE5 with (−)-evodiamine (**e**). In (**c**–**e**), the closer the color is to red, the higher the value of the B-factor is.

We calculated the RMSF to study how evodiamine binding affects protein fluctuations. In Figure 12a, we show the RMSF of PDE5 and the complex of PDE5 from 50 ns to 200 ns, when the change over time of whole RMSD becomes small. RMSF results showed that the fluctuations at residues 606–614 and 760–830 were larger in the (−)-evodiamine complex than in PDE5 and its (+)-evodiamine complex. Figure 12c–e shows the average structures at 50–200 ns. In these snapshots, the color changes depending on the value of the B-factor calculated from the RMSF, with the larger value being closer to red. The residue of 607–612 had two states (Figure 12b). Therefore, the peak of 607–612 is not a fluctuation but large RMSF value (Figure 12a). 

### 3.5. Hydrogen Bonds between PDE5 and Evodiamine

**Figure 13:**
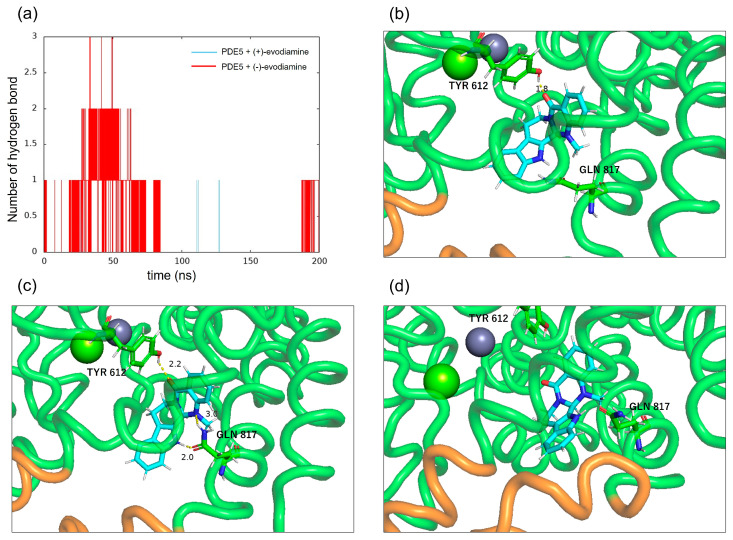
Intermolecular hydrogen bonds between PDE5 and evodiamine. (**a**) Number of hydrogen bonds between PDE5 and evodiamine. (**b**) Hydrogen bond between the oxygen atom of (−)-evodiamine and the hydroxy group of Tyr612. (**c**) Hydrogen bonds between the two nitrogen atoms of evodiamine and the side of Gln817. (**d**) Hydrogen bond between the nitrogen atom of evodiamine and the carbonyl group of Gln817.

To confirm the binding of PDE5 to evodiamine, the number of intermolecular hydrogen bonds was calculated. Figure 13a shows the time evolution of the number of intermolecular hydrogen bonds formed between PDE5 and evodiamine. There are up to three hydrogen bonds between PDE5 and evodiamine (Figure 13a). A maximum of three bonds are formed from the beginning of the simulation up to 50 ns, but after that, no hydrogen bonds were formed for a long time. First, evodiamine forms a hydrogen bond with Tyr612 (H-loop) in the Q pocket (Figure 13b). It then forms a hydrogen bond with Gln817 (M-loop) and is fixed in orientation (Figure 13c). In particular, the bidentate hydrogen bond is similar to the binding of sildenafil with Gln817, as shown in Figure 3b. Finally, a multi-membered ring of (−)-evodiamine is sandwiched between the M-loops in Figure 13d. 

## 4. Discussion

**Figure 14:**
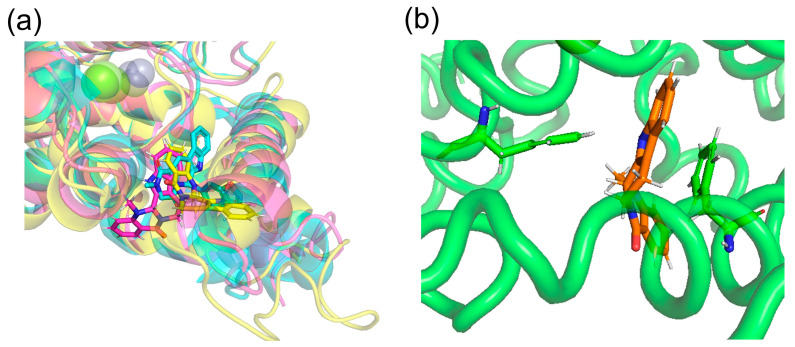
(**a**) (+)-evodiamine (magenta), (−)-evodiamine (yellow), and sildenafil (cyan). (**b**) PDE5 and (+)-evodiamine at 148 ns. Ligand is sandwiched between two phenylalanines.

Docking simulations by SwissDock showed that (+)-evodiamine was not in the pocket, contrary to the case of tadalafil, which is an inhibitor. However, in the MD simulation, the multimembered ring moved into the pocket (Figure 14a). In this case, the multimembered ring of (+)-evodiamine was in the same position as the multimembered ring of tadalafil. There is a multi-membered ring between Phe786 and Phe820 in the pocket (Figure 14b). Therefore, a flat structure is considered to be more likely to fit into the pocket. Comparison of RMSDs showed that the complexes have a less altered M-loop structure than PDE5 alone. Then, PDE5 with (+)-evodiamine complex suggested that binding of PDE5 to the ligand constructed the M-loop. 

On the other hand, (−)-evodiamine was found to have two docking sites, a reaction center pocket shown with sildenafil (PDBID: 1UDT, Figure 14a), and an allosteric pocket, which was found by T. Zhang et al. [33]. In the results, the ΔG for the reaction center pocket and allosteric pocket are -7.5681434 and −7.189539 kcal/mol, respectively, suggesting that they do not selectively bind to either pocket. In the allosteric pocket, (+)-evodiamine is not pocketed in the simulation. In the case of (−)-evodiamine, we have performed an MD simulation starting with this molecule in the reaction center pocket. In the future, MD simulation starting in the allosteric pockets should be performed.

Comparing the PDE5 and both PDE5 complexes, we found differences in the conformational change of the M-loop in the (−)-evodiamine complex. The planar structure of evodiamine is less stable than the tilted structure. The (−)-evodiamine is ring-inverted with the start of MD. This dihedral angle of the side-chain of Phe786 near the M-loop was found to be different from the case of other models (PDE5 or PDE5 with (+)-evodiamine). This is due to the fact that the (−)-evodiamine was guided by hydrogen bonds and moved to enter between the M-loop. It has been suggested that existing inhibitors can also disrupt the loop structure [20], and our results support this. The binding of nitrogen of evodiamine to Gln817 is essential for this hydrogen bond. 

Tadalafil is known to have stronger inhibitory activity than sildenafil [34]. However, unlike sildenafil, it is reported to form only one hydrogen bond between Gln817 and the NH of the indole ring, without forming a bidentate hydrogen bond [35]. Relying on this strong hydrogen bond, tadalafil is stabilized in the active site. The tadalafil backbone is also stiffer than that of sildenafil, suggesting a higher affinity for tadalafil with less entropy loss [23]. Evodiamine has a similar main backbone structure to tadalafil than sildenafil, but there are differences in the formation of bidentate hydrogen bonds. The main backbone structure of evodiamine is similar to that of tadalafil. This simulation confirms that in (−)-evodiamine, the ring inversion changes the conformation of the methyl group of this N14, exposing N14 and forming a bidentate hydrogen bond with PDE5. Our quantum mechanical calculations show that the conformation forming this bidentate hydrogen bond is stable. The methyl group of (−)-evodiamine was positioned in a pseudo-equatorial position. Our results suggest that evodiamine forms a bidentate hydrogen bond similar to that of sildenafil, an existing PDE5 inhibitor. It is possible to consider using a different substituent for this methyl group to create a derivative with stronger binding strength.

Evodiamine binding increased the RMSF of the M-loop and H-loop. The M-loop is known to be involved in the selective inhibitory activity of several families of PDEs [17]. The present results did not confirm the binding of the two loop structures. However, the larger RMSF of these structures may have an effect on the selective inhibitory activity. Future studies with other proteins such as PDE4 should be interesting. A novel method for predicting ligand–protein binding affinity from conformational fluctuations of BRD4 protein by machine learning is reported [36]. It is not the magnitude of the RMSF that is important, but the residue where variation occurs. It is necessary to analyze the characteristics of the RMSF in MD simulations of various inhibitors, including evodiamine in this study. In the future, detailed calculations of coupling free energies should be performed. Therefore, MM/GBSA calculations should be considered along with new MD simulations.

## 5. Conclusions

PDE5 is a potential target protein for the treatment of Alzheimer’s disease. We performed MD simulations of the PDE5 complexes by docking evodiamine, one of the indole alkaloids in Euodiae Fructus, to PDE5. Binding with (+)-evodiamine increased *R*_g_ overall, but rather decreased it in the M-loop. This suggests that the M-loop would possibly be stabilized by the ligand. The results showed that the binding of (−)-evodiamine increased the RMSD and *R*_g_ of PDE5. The conformational change in evodiamine was consistent with a change in the C_5_-N_6_-C_13b_-C_13a_ dihedral angle. This indicates that both evodiamine stereoisomers are ring-inverted and stable conformationally. In (−)-evodiamine, the conformation of the M-loop changed significantly. In the PDE5 complexes, an increase in the RMSF of the M-loop was observed, which was attributed to the ring inversion of (−)-evodiamine in the pocket and the associated change in the shape of the M-loop. This result suggests that (−)-evodiamine may have inhibitory activity. Moreover, the addition of functional groups to allow hydrogen bonding at stable conformations may be an indicator for designing more potent inhibitors.

## Figures and Tables

**Table 1:** The systems of MD simulation for PDE5 and complexes.

	PDE5	PDE5 + (+)-Evodiamine	PDE5 + (−)-Evodiamine
Water	60,898	70,097	70,067
Zn^2+^	1	1	1
Mg^2+^	1	1	1
Na^+^	5	5	5
Box size (nm^3^)	1892.4	2174.3	2175.8
Temperature (K)	300	300	300

## Data Availability

The data presented in this study are available upon request from the corresponding author.
